# AS-IV enhances the antitumor effects of propofol in NSCLC cells by inhibiting autophagy

**DOI:** 10.1515/med-2023-0799

**Published:** 2023-09-25

**Authors:** Jintao Liu, Long Chen, Jialing Zhang, Xiaopan Luo, Yingyi Tan, Shaojie Qian

**Affiliations:** Center for Rehabilitation Medicine, Department of Anesthesiology, Zhejiang Provincial People’s Hospital (Affiliated People’s Hospital, Hangzhou Medical College), Hangzhou, Zhejiang, China; The Second School of Clinical Medicine, Zhejiang Chinese Medical University, Hangzhou, Zhejiang, China; Rehabilitation Medicine Center, Department of Nursing, Zhejiang Provincial People’s Hospital (Affiliated People’s Hospital, Hangzhou Medical College), Hangzhou, Zhejiang, China; Center for Rehabilitation Medicine, Department of Anesthesiology, Zhejiang Provincial People’s Hospital (Affiliated People’s Hospital, Hangzhou Medical College), No. 158 Shangtang Road, Gongshu District, Hangzhou, Zhejiang, China

**Keywords:** non-small cell lung cancer, anesthetics, propofol, astragaloside IV, autophagy

## Abstract

Non-small cell lung cancer (NSCLC) is one of the most lethal malignant tumors. It has been shown that the general anesthetic agents, propofol and astragaloside IV (AS-IV) both exert antitumor effects in NSCLC. However, the effects of the combination of propofol with AS-IV in NSCLC remain unclear. Cell counting kit-8, and EdU and Transwell assays were performed to evaluate NSCLC cell viability, proliferation, and migration. Cell apoptosis and autophagy were observed by flow cytometric analysis and TUNEL and LC3 staining, respectively. AS-IV notably enhanced the anti-proliferative, pro-apoptotic, and anti-migratory properties of propofol in NSCLC cells. Moreover, AS-IV remarkably facilitated the anti-autophagy effect of propofol in NSCLC cells by downregulating LC3, Beclin 1, and ATG5. Significantly, the pro-apoptotic ability of the AS-IV/propofol combination in NSCLC cells was further enhanced by the autophagy inhibitor 3-MA, suggesting that autophagy plays a tumor-promoting role in NSCLC cells. Collectively, AS-IV could facilitate the antitumor abilities of propofol in NSCLC cells by inhibiting autophagy. These findings may be beneficial for future studies on the use of AS-IV and propofol for the treatment of NSCLC.

## 1 Introduction

Non-small cell lung cancer (NSCLC) is one of the most common malignancies, representing 80% of total lung cancer cases [1]. The overall survival rate of NSCLC is poor due to delays in diagnosis and metastasis potential of this disease [2,3]. Recently, even with aggressive treatment protocols, including complete surgical resection, radiotherapy, immunotherapy, and chemotherapy, relapse was developed in the majority of NSCLC patients (25–70%) within 5 years [4–6]. Therefore, discovering novel efficient therapeutic approaches for NSCLC is urgently needed.

Local anesthetics are extensively applied in clinical cancer surgeries [7,8]. Recently, evidence has shown that anesthetics also exhibit antitumor properties in multiple cancers, including NSCLC [9–11]. Propofol, a general anesthetic agent administered during surgery, has been found to suppress the progression of NSCLC [12]. Zhang et al. found that propofol could decrease NSCLC cell proliferation, migration, and invasion [13]. Meanwhile, propofol was able to enhance the sensitivity of lung cancer cells to cisplatin in NSCLC [14].

It has been shown that traditional Chinese medicine combined with antitumor drugs could achieve favorable effects for cancer treatment [15,16]. Astragalus membranaceus, a type of traditional Chinese medicine, possesses anti-inflammatory, antioxidative, and antitumor effects [17,18,19]. Astragaloside IV (AS-IV) is a main active component isolated from Astragalus membranaceus [20]. Jia et al. found that AS-IV could reduce NSCLC cell proliferation and migration by inhibiting Akt/GSK-3β signaling [21]. In addition, AS-IV could improve the response of NSCLC cells to cisplatin by inhibiting autophagy [22]. However, the role of the combination of AS-IV and propofol in NSCLC remains unclear. Therefore, we aimed to explore the antitumor activities of AS-IV combined with propofol on NSCLC cells. In this study, we found that AS-IV could enhance the pro-apoptotic and anti-migratory activities of propofol in NSCLC cells. These findings may be beneficial for future studies on the use of AS-IV and propofol for the treatment of NSCLC.

## 2 Materials and methods

### 2.1 Cell culture

Normal lung epithelial cells (BEAS2B) and NSCLC cell lines A549 and NCI-H1299 were obtained from Procell Life Science & Technology Co., Ltd. A549 and NCI-H1299 cells were maintained in complete medium consisting of DMEM (Thermo Fisher Scientific), 0.1% penicillin‒streptomycin, and 10% fetal bovine serum (FBS, Thermo Fisher Scientific, category number: 26010-074) at 37°C in a humidified atmosphere of 5% CO_2_. Cells were maintained in a 75 cm^2^ culture flask for culture. Cells were subcultured every 3 days with a maximum of passages not exceeding 25. When the cells grew to 70% confluence, they were collected and used for subsequent experiments.

### 2.2 Cell counting kit-8 (CCK-8) assay

BEAS2B or NSCLC cells (1  ×  10^4^ cells/well) were plated into 96‐well plates overnight. Next NSCLC cells were treated with AS-IV (0, 5, 10, 20, or 40 ng/mL) [21] and/or propofol (0, 2.5, 5, 10, or 20 μg/mL) [23] for 48 h. After that, each well was supplemented with CCK-8 reagent (10 µL; Beyotime), and the cells were then incubated for 2 h. Finally, the absorbance at 450 nm was measured with a microplate reader (DR-200Bs, Diatek). AS-IV and propofol were obtained from Sigma-Aldrich (PHL89377 and Y0000016, Sigma-Aldrich, St Louis, USA).

### 2.3 EdU staining assay

The proliferative capacity of A549 and NCI-H1299 cells was assessed by EdU staining assay. The EdU assay was conducted using the Cell-Light EdU DNA cell proliferation kit (RiboBio). Briefly, NSCLC cells were treated with EdU solution (50 µM) for 2 h. After fixation with 4% paraformaldehyde (PFA), cells were stained with Apollo reagent for 30 min in darkness. The nuclear DNA was then stained with Hoechst 33342. Finally, EdU-positive cells were observed under a fluorescence microscope (OLYMPUS).

### 2.4 Flow cytometric analysis

Flow cytometry analysis was used to evaluate the apoptosis of A549 and NCI-H1299 cells. The cells were collected, and the apoptotic cells were identified with an annexin V-FITC cell apoptosis detection kit (product No. C1063; Biotech Research Institute) according to the manufacturer’s protocol. In short, 1 × 10^6^ cells were resuspended in 1 mL binding buffer, incubated with 5 µL of Annexin V-FITC for 15 min, and then incubated at 4°C with 5 µL of PI for 5 min in the dark. Fluorescence signals were collected by FACScan flow cytometry (Beckman Coulter, Inc. and then analyzed by FlowJo 8.7.1 software (FlowJo LLC).

### 2.5 TUNEL staining assay

The TUNEL assay was conducted using the *In Situ* Cell Death Detection Kit (Roche). Briefly, NSCLC cells were incubated with the TUNEL working solution for 1 h in darkness. The nuclear DNA was then stained with DAPI for 30 min. Finally, the apoptotic cells were captured under a fluorescence microscope.

### 2.6 Transwell migration assay

NSCLC cells (2  ×  10^4^ cells) suspended in serum-free DMEM were added to the upper compartment of 24-well migration chambers (Corning). Meanwhile, the lower compartment was filled with 500 µL of DMEM containing 10% FBS as the attracting agent. Cells in the upper chamber migrating through the polycarbonate membrane into the lower chamber with high nutritional content were considered to have high migration ability. Next 0.1% crystal violet was used to stain the migrated cells on the undersurface of the lower chamber at 24 h. Finally, the migrated cells were captured with a microscope.

### 2.7 Immunofluorescence assay

About 2 × 10^4^ NSCLC cells were inoculated into a 24-well plate and fixed at room temperature with 4% PFA for 20 min. PBS containing 1% Triton X-100 was then added to permeate for 20 min. The cells were then incubated with anti-LC3 (ab192890, 1:1,000, Abcam) specific rabbit monoclonal antibody at 4°C overnight. The goat anti-rabbit IgG H&L (Alexa Fluor^®^ 647) secondary antibody (ab150079, 1:1,000, Abcam) was then incubated at room temperature in darkness for 2 h. The nuclei were stained with DAPI. Subsequently, the LC3-positive cells were observed using a fluorescence microscope.

### 2.8 Western blot assay

Total protein was extracted from the cells using RIPA lysis buffer. Total proteins were quantified by the BCA protein assay kit (ASPEN). Next proteins (20 μg/lane) were dissolved by 10% SDS-PAGE and then electrotransferred onto a PVDF membrane (Millipore). After that, the membrane was incubated with specific primary antibodies, including anti-Beclin 1 (ab207612, 1:2,000, Abcam), anti-ATG5 (ab108327, 1:1,000, Abcam), anti-ERK (ab184699, 1:10,000, Abcam), anti-p-ERK (ab201015, 1:1,000, Abcam), anti-Bcl-2 (ab32124, 1:1,000, Abcam), anti-cleaved caspase 3 (ab32042, 1:500, Abcam), and anti-β-actin (ab6276, 1:5,000, Abcam), at 4°C overnight. Following incubation with the corresponding secondary antibody Goat Anti-Rabbit IgG H&L (HRP) (ab7090, 1:5,000, Abcam), the protein blots were developed by ECL reagent. Band intensity was measured using ImageJ software (ImageJ, NIH).

### 2.9 Statistical analyses

All data were repeated at least three times independently. Data are shown as the mean value ± standard deviation and analyzed with Graphpad Prism 7.0. Statistical analysis was performed by one-way analysis of variance (ANOVA) followed by Tukey’s test. *p* values <0.05 were considered statistically significant.

## 3 Results

### 3.1 AS-IV enhanced the cytotoxic effect of propofol in NSCLC cells

**Figure 1:**
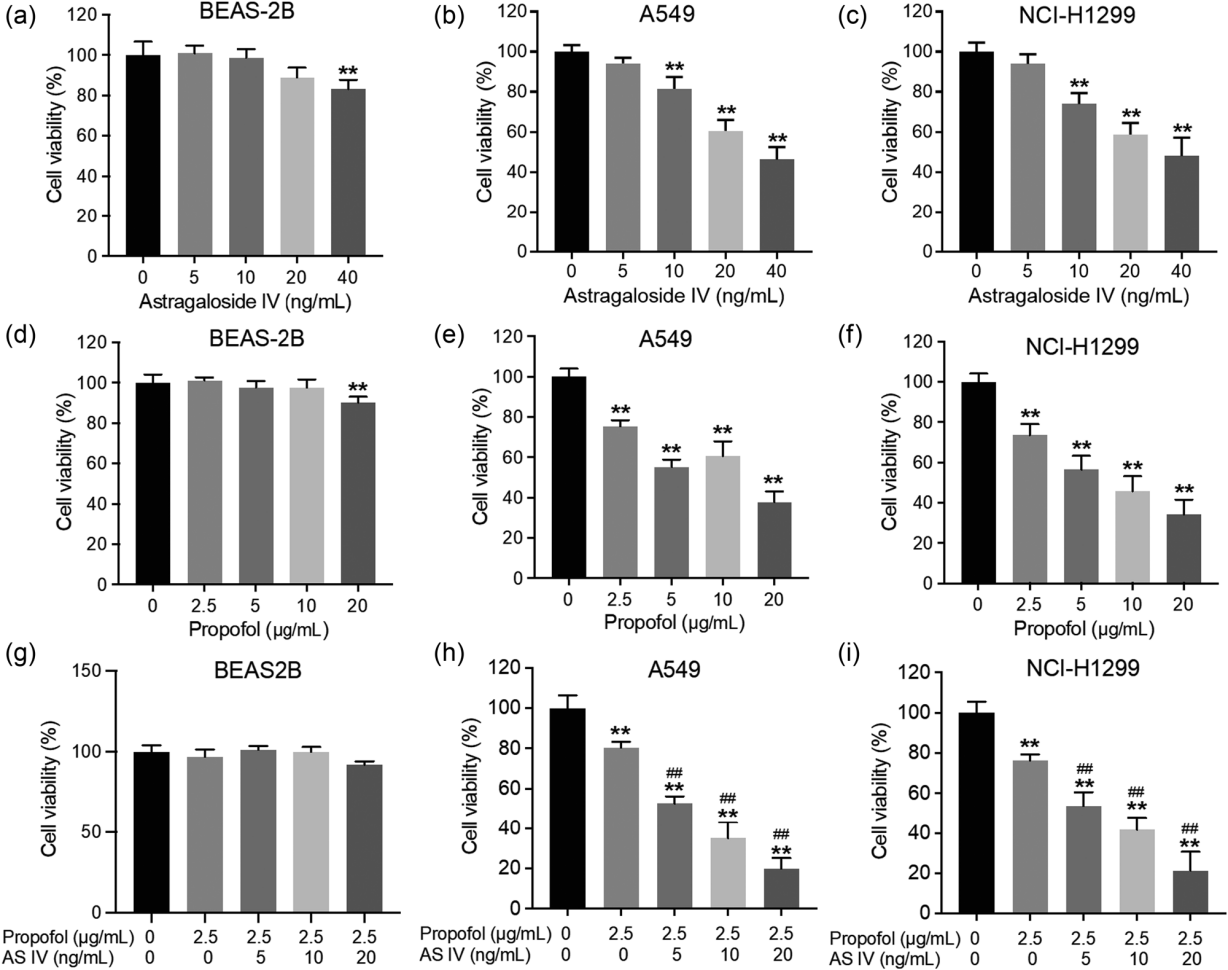
AS-IV enhanced the cytotoxic effect of propofol in NSCLC cells. (a) BEAS2B, (b) A549, and (c) NCI-H1299 cells were treated with AS-IV (0, 5, 10, 20, or 40 ng/mL) for 48 h to evaluate cell viability using CCK-8 assay. (d) BEAS2B, (e) A549, and (f) NCI-H1299 cells were treated with propofol (0, 2.5, 5, 10, or 20 μg/mL) for 48 h. (g) BEAS2B, (h) A549, and (i) NCI-H1299 cells were treated with propofol (2.5 μg/mL) and AS-IV (0, 5, 10, or 20 ng/mL) for 48 h. The 2.5 μg/mL propofol and 5 ng/mL AS-IV were utilized in the following experiments. **p < 0.01 vs control group; ^##^ *p* < 0.01 vs propofol (2.5 μg/mL) treatment group.

To investigate the role of AS-IV and propofol in NSCLC cells, CCK-8 assay was conducted to determine the effects of AS-IV and propofol on the viability of BEAS2B, A549, and NCI-H1299 cells. As shown in Figure 1a, no significant change in BEAS2B cell viability was observed when the concentration of AS-IV was less than 20 ng/mL. As shown in Figure 1b and c, AS-IV (10, 20, or 40 ng/mL) notably inhibited the viability of NSCLC cells. The viability of both A549 and NCI-H1299 cells at 40 ng/mL AS-IV was reduced to below 60%. Figure 1d showed that compared with the control group, there was no statistical significance in cell viability of BEAS-2B cells treated with propofol when the concentration was 2.5, 5, 10 μg/mL. Compared with the control group, the cell viability decreased when the concentration of propofol was 20 μg/mL, which had statistical significance. Propofol (2.5, 5, 10, or 20 μg/mL) markedly reduced the viability of NSCLC cells (Figure 1e and f). Furthermore, 2.5 μg/mL propofol significantly decreased NSCLC cell viability and exhibited approximately 20% growth inhibition. The viability of both A549 and NCI-H1299 cells at 20 μg/mL propofol was reduced to below 50%, but it had no significant effect on the cell survival rate of BEAS2B. In particular, the combination of propofol (2.5 μg/mL) and AS-IV (5 ng/mL) markedly reduced NSCLC cell viability and exhibited approximately 50% growth inhibition (Figure 1g–i). Therefore, 2.5 μg/mL propofol and 5 ng/mL AS-IV were utilized in the following experiments. Collectively, AS-IV could enhance the cytotoxic effect of propofol in NSCLC cells.

### 3.2 AS-IV enhanced the anti-proliferative, pro-apoptotic, and anti-migratory properties of propofol in NSCLC cells

**Figure 2:**
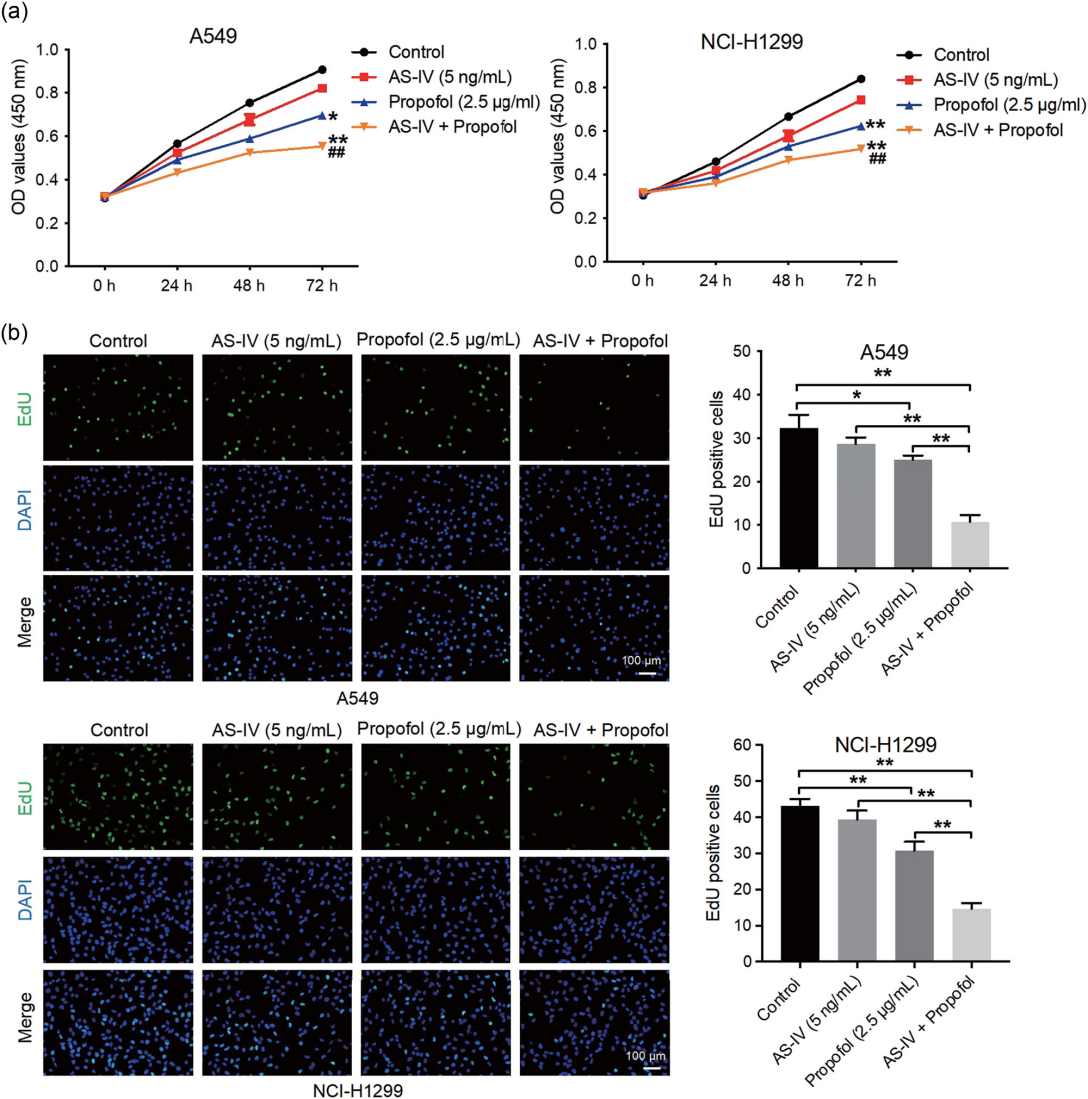
AS-IV enhanced the anti-proliferative property of propofol in NSCLC cells. A549 and NCI-H1299 cells were treated with AS-IV (5 ng/mL) and/or propofol (2.5 μg/mL) for 48 h. Cell proliferation was assessed by CCK-8 (a) and EdU (b) staining assays (200×). The combination of AS-IV with propofol further inhibited the proliferation of NSCLC cells compared to the propofol treatment alone. *p < 0.05, **p < 0.01 vs control group; ^##^ *p* < 0.01 vs propofol (2.5 μg/mL) treatment group.

**Figure 3:**
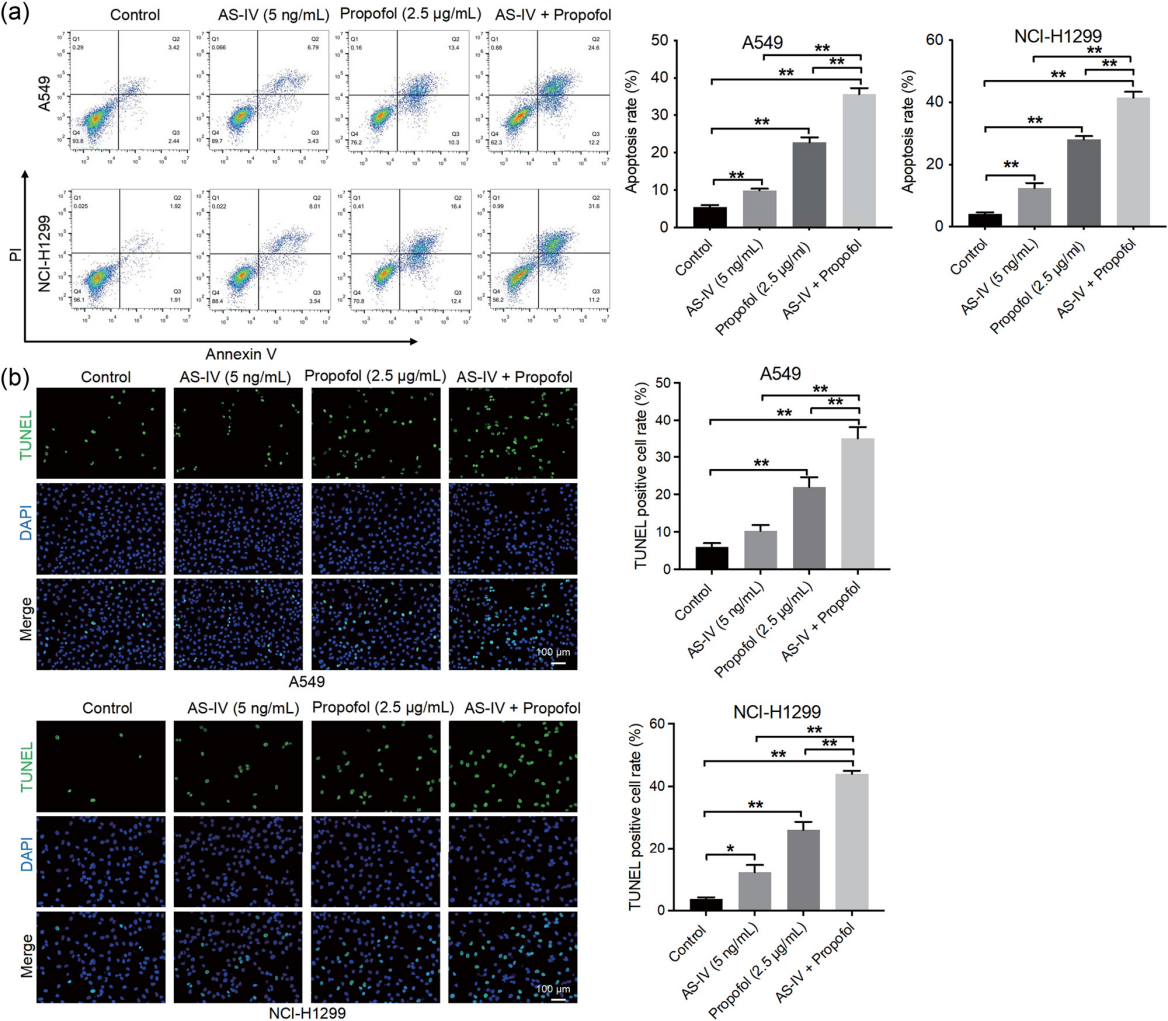
AS-IV enhanced the pro-apoptotic property of propofol in NSCLC cells. A549 and NCI-H1299 cells were treated with AS-IV (5 ng/mL) and/or propofol (2.5 μg/mL) for 48 h. Cell apoptosis was assessed by flow cytometric analysis (a) and TUNEL assay (b) (200×). AS-IV further strengthened propofol-induced NSCLC cell apoptosis. *p < 0.05, **p < 0.01.

**Figure 4:**
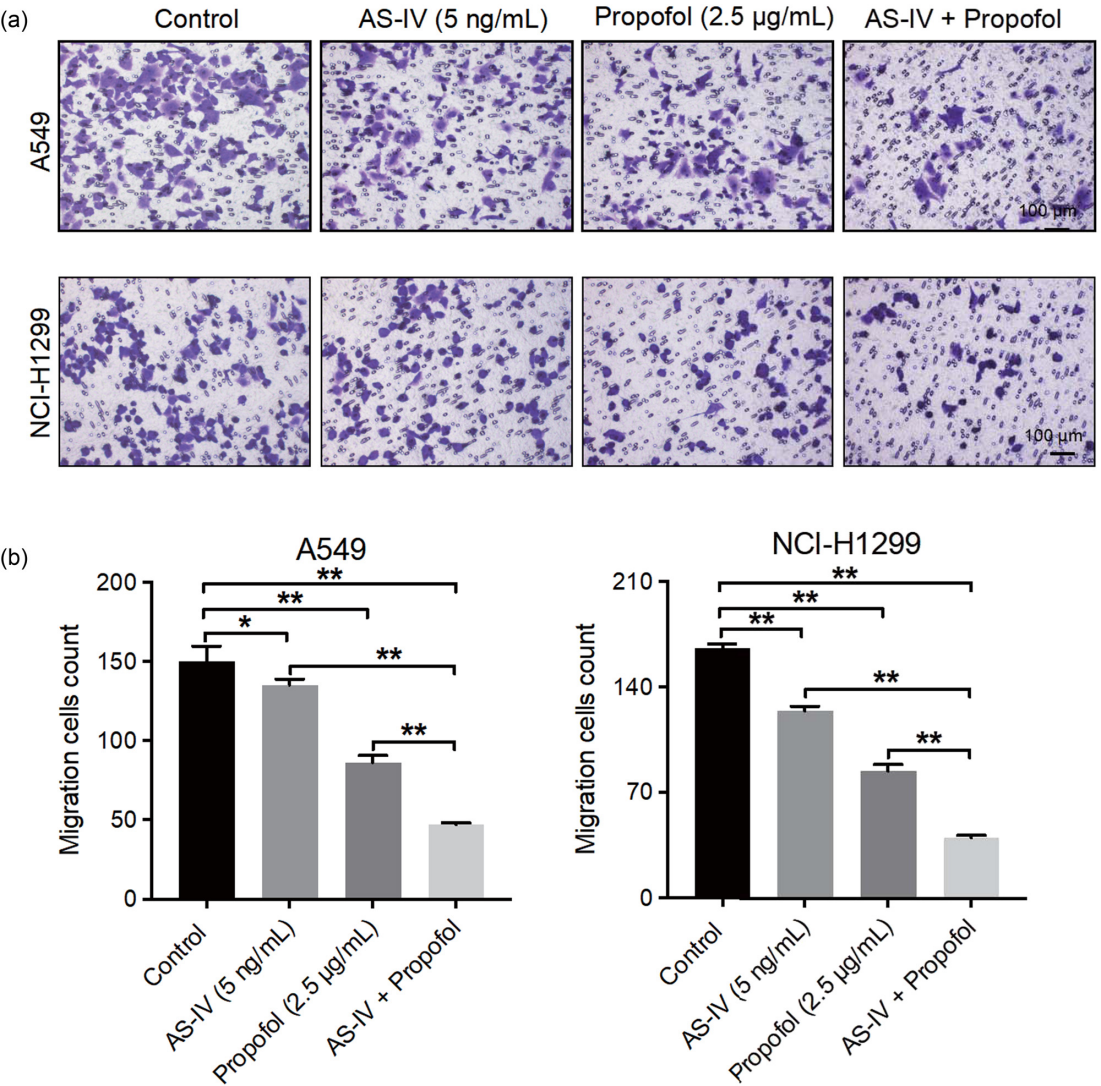
AS-IV enhanced the anti-migratory property of propofol in NSCLC cells. (a) A549 and (b) NCI-H1299 cells were treated with AS-IV (5 ng/mL) and/or propofol (2.5 μg/mL) for 48 h. Cell migration was assessed by Transwell assay (200×). The combination of AS-IV and propofol further reduced the migratory ability of NSCLC cells compared with propofol treatment alone. *p < 0.05, **p < 0.01.

Next we explored the effects of AS-IV and propofol on the proliferation, apoptosis, and migration of NSCLC cells. As indicated in Figure 2a and b, propofol (2.5 μg/mL) significantly suppressed NSCLC cell proliferation. As expected, the combination of AS-IV with propofol further inhibited the proliferation of NSCLC cells (the proportion of Edu-positive cells was decreased by 2-fold) compared to that of cells in the propofol treatment alone group (Figure 2a and b). Additionally, propofol treatment obviously resulted in increased NSCLC cell apoptosis (Figure 3a and b). Interestingly, AS-IV further strengthened propofol-induced NSCLC cell apoptosis (cell apoptosis above 35%) (Figure 3a and b). Furthermore, propofol or AS-IV treatment led to a remarkable decrease in NSCLC cell migration (Figure 4a and b). Meanwhile, the combination of AS-IV and propofol further reduced the migratory ability of NSCLC cells (the number of migrating cells decreased by more than 3-fold) compared to the propofol treatment alone group (Figure 4a and b). Collectively, AS-IV enhanced the anti-proliferative, pro-apoptotic, and anti-migratory properties of propofol in NSCLC cells.

### 3.3 Combination of AS-IV and propofol suppressed NSCLC cell autophagy and ERK1/2 signaling

**Figure 5:**
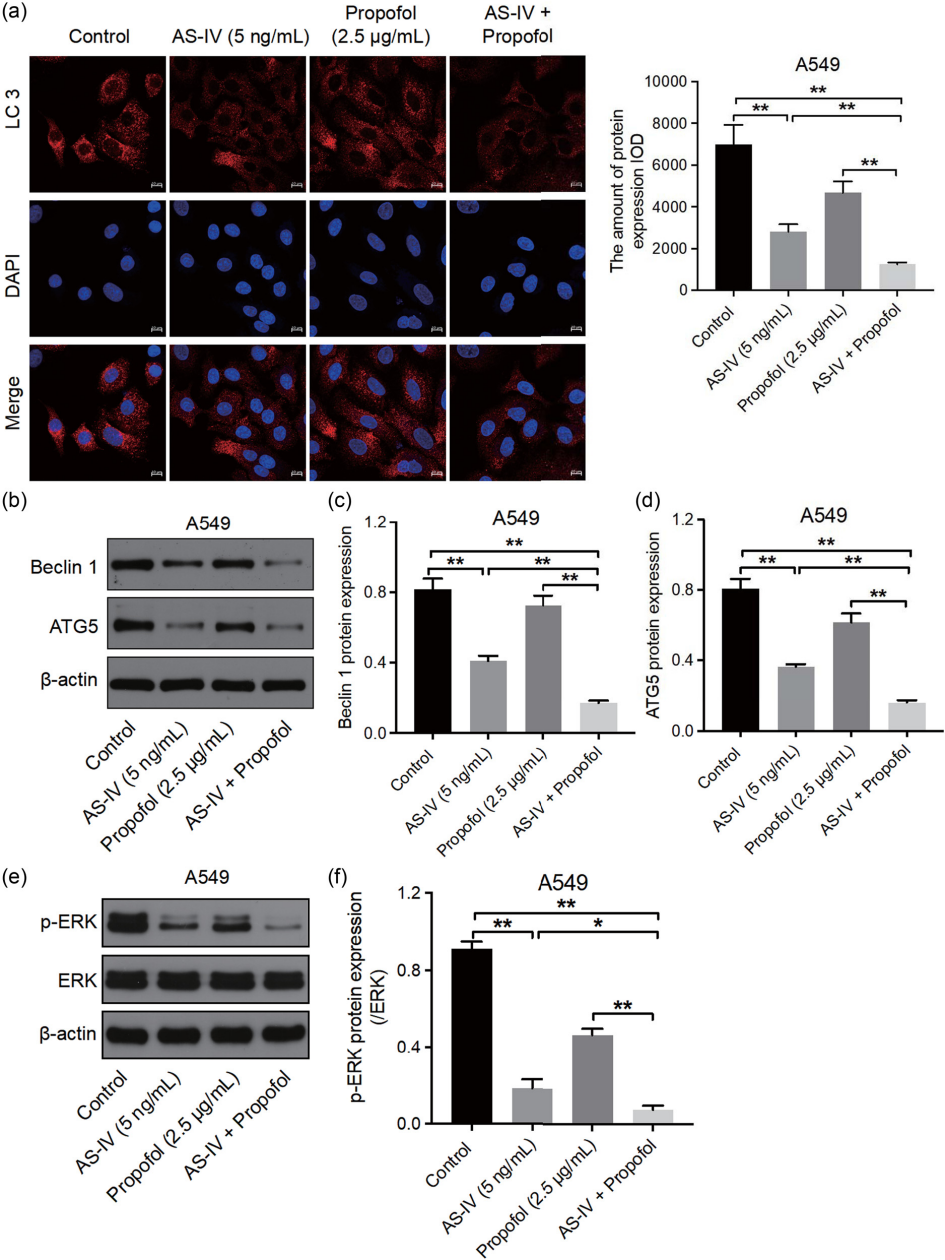
The combination of AS-IV and propofol suppressed NSCLC cell autophagy. A549 cells were treated with AS-IV (5 ng/mL) and/or propofol (2.5 μg/mL) for 48 h. (a) LC3 levels in A549 cells were detected by IF staining assay (400×). (b)–(f) Western blot assay was applied to determine Beclin 1, ATG5, and p-ERK/ERK levels in A549 cells. IF, Immunofluorescence. **p < 0.01.

Autophagy is a highly conserved cellular proteolysis process that plays an important role in cancer development [24]. Thus, we next explored whether AS-IV and propofol could affect NSCLC cell autophagy. As shown in Figure 5a–f, AS-IV (5 ng/mL) group and propofol (2.5 μg/mL) group significantly reduced LC3, Beclin 1, ATG5, and p-ERK/ERK levels in NSCLC cells. Interestingly, the combination of AS-IV and propofol further down-regulated LC3, Beclin 1, ATG5, and p-ERK/ERK levels in NSCLC cells. In summary, the combination of AS-IV and propofol suppressed NSCLC cell autophagy.

### 3.4 AS-IV combined with propofol triggered NSCLC cell apoptosis by inhibiting autophagy

**Figure 6:**
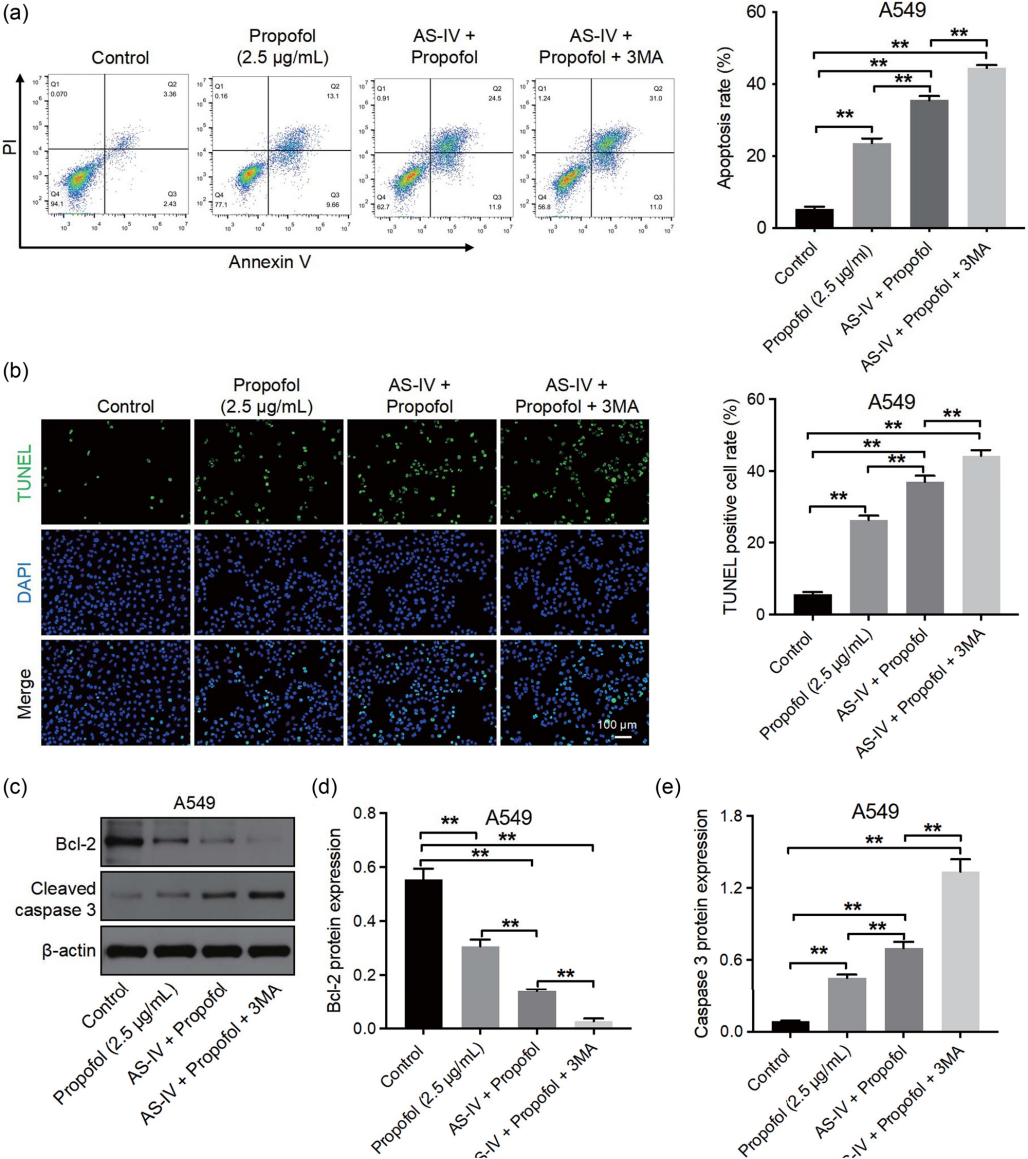
The combination of AS-IV and propofol triggered NSCLC cell apoptosis by inhibiting autophagy. A549 cells were pretreated with 5 mM 3-MA prior to 12 h and then treated with propofol or propofol plus AS-IV for 48 h. Cell apoptosis was assessed by flow cytometric analysis (a) and TUNEL assay (b) (×200×). (c)–(e) Western blot assay was applied to determine Bcl-2 and cleaved caspase 3 levels in A549 cells. Combination of AS-IV and propofol could trigger NSCLC cell apoptosis by inhibiting autophagy. *p < 0.05, **p < 0.01.

Since the AS-IV/propofol combination could induce NSCLC cell apoptosis and suppress cell autophagy, we then focused on the interaction between apoptosis and autophagy in NSCLC cells. The pro-apoptotic effect of the AS-IV/propofol combination on NSCLC cells was further enhanced by treatment with the autophagy inhibitor 3-MA, as shown by the decreased level of Bcl-2 and increased level of cleaved caspase 3 (Figure 6a–e). Collectively, the combination of AS-IV and propofol could trigger NSCLC cell apoptosis by inhibiting autophagy.

## 4 Discussion

Recently, Chinese traditional medicine has been used in the treatment of NSCLC [25,26]. AS-IV isolated from Astragalus membranaceus has been recognized to exert anticancer effects in NSCLC [21]. In this research, we found that AS-IV could enhance the antitumor effects of propofol in NSCLC cells by suppressing autophagy.

Propofol, an anesthesia drug, has been proven to act as an antitumor agent in multiple cancers [27,28]. For example, propofol inhibits the development of bladder cancer [29] and colon cancer [30] by regulating miR-145-5p or JAK2/STAT3 signaling pathway. Propofol increases miR-486-5p levels in NSCLC cells and xenograft tumor tissues in a N6-methyladenosine (m6A) dependent manner, thereby inactivating the Ras associated protein 1 (RAP1)-NF-kappaB (NF-κB) axis to increase cisplatin sensitivity in NSCLC [23]. Propofol inhibits the development of NSCLC by inhibiting the circ-RHOT1/miR-326/Prognostic Significance of Forkhead Box M1 (FOXM1) [13] axis and miR-21/PTEN/AKT [12] axis. In addition, propofol could trigger NSCLC cell apoptosis by inactivating ERK1/2 signaling and upregulating p53-upregulated modulator of apoptosis [31]. These results demonstrate that propofol can function as a tumor suppressor in cancer through miRNA, JAK2/STAT3, RAP1/NF-κB, PTEN/AKT, and ERK1/2 signaling pathways. On the basis of these previous studies, our results showed that propofol treatment resulted in a significant decrease in NSCLC cell proliferation and migration and an increase in cell apoptosis. These results further confirmed the antitumor effects of propofol in NSCLC.

Recently, combination therapy has attracted increasing attention in cancer treatment due to the advantages of reduced toxicity, synergistic antitumor effects, and diminished acquired resistance [32,33,34]. Propofol regulates Wnt/β-Catenin, HIF-1 signaling pathway, circ-ERBB2/miR-7-5p/FOXM1 axis, inhibits NSCLC cell proliferation, invasion, and glycolysis, and accelerates cell apoptosis [14,35,36]. AS-IV inhibits AMPK signaling pathway, Akt/GSK-3β/β-Catenin, endoplasmic reticulum stress signaling pathway, and autophagy signaling pathway and evidence in previous studies also proved AS-IV inhibits the proliferation of NSCLC and promotes apoptosis [21,22,37]. This study found that the combination of propofol and AS-IV could further inhibit ERK1/2 signaling in NSCLC cells. Therefore, the combined action of propofol and AS-IV can synergistically act on multiple signal pathways, thereby inducing apoptosis in lung cancer cells. The combination of propofol and sevoflurane remarkably suppressed the migration and invasion of lung adenocarcinoma cells compared to single drugs [38]. The combination of propofol and paclitaxel obviously induced apoptosis in prostatic cancer cells compared to paclitaxel alone [39]. In addition, AS-IV and curcumin synergistically inhibited tumor growth in hepatocellular carcinoma [40]. These results predict that the therapeutic effect of the combination may be more significant than that of the single medication. Whether AS-IV would produce synergistic effects with propofol in NSCLC has not been reported. While the present study conducted a preliminary exploratory study of this blank, in this study, we found that AS-IV could strengthen the antitumor effects of propofol in NSCLC cells. Meanwhile, the combination of AS-IV and propofol exhibited broad antitumor activity compared to single drug treatment.

Autophagy is a self-degradative system that can exert tumor-promoting or antitumor effects in different contexts [41,42]. Propofol could enhance tumor sensitivity to cisplatin in gastric cancer by inhibiting autophagy [43]. In addition, AS-IV was able to induce vulvar squamous cell carcinoma cell apoptosis and autophagy by regulating TGF-β/Smad signaling [44]. Moreover, AS-IV was found to sensitize NSCLC cells to cisplatin treatment by inhibiting autophagy [22]. In this study, AS-IV significantly suppressed NSCLC cell autophagy, and propofol slightly suppressed NSCLC cell autophagy. As expected, the combination of AS-IV and propofol further inhibited NSCLC cell autophagy compared to single drug treatment by inhibiting LC3, Beclin1, and ATG5. In addition, inhibition of autophagy by 3-MA further increased the effect of combination-induced NSCLC cell apoptosis. These results showed that autophagy played a tumor-promoting effect in NSCLC cells. Collectively, combined AS-IV with propofol was able to trigger NSCLC cell apoptosis by inhibiting autophagy.

However, there are some limitations to this study. In this study, the effects of AS-IV and propofol via autophagy on the proliferation, migration, and apoptosis of NSCLC cells were investigated only at the cellular level *in vitro* and were not further verified at the *in vivo* level. In the future, it is necessary to construct an animal model of subcutaneous tumor of NSCLC to further study the anti-tumor effect of AS-IV combined with propofol. The lack of clinical trial data is also one of the limitations of this study. In addition, in this study, coadministration of AS-IV and propofol inhibited autophagy in lung cancer cells by further downregulating the level of the autophagy protein Beclin1, but the specific molecular mechanism still needs further investigation. In addition to that, we only explored the antitumor effects of propofol in NSCLC cells, future studies are suggested to explore the combined effect of other anesthetics, such as sevoflurane and dexmedetomidine, with AS-IV in NSCLC cells. These limitations need to be carefully addressed before this study can be translated into clinical practice.

## 5 Conclusion

In conclusion, AS-IV enhanced the antitumor effects of propofol in NSCLC cells by inhibiting autophagy. These findings might pave the way for the application of AS-IV and propofol in NSCLC in the future.

## References

[1] Liu JC, Narva S, Zhou K, Zhang W. A review on the antitumor activity of various nitrogenous-based heterocyclic compounds as NSCLC inhibitors. Mini Rev Med Chem. 2019;19(18):1517–30. 10.2174/1389557519666190312152358 30864519

[2] Zhou F, Sun J, Ye L, Jiang T, Li W, Su C, et al. Fibronectin promotes tumor angiogenesis and progression of non-small-cell lung cancer by elevating WISP3 expression via FAK/MAPK/HIF-1alpha axis and activating wnt signaling pathway. Exp Hematol Oncol. 2023;12(1):61. 10.1186/s40164-023-00419-w PMC10355078 37468964

[3] Hou F, Hou Y, Sun XD, Lv J, Jiang HM, Zhang M, et al. Establishment of a prognostic risk prediction model for non-small cell lung cancer patients with brain metastases: a retrospective study. PeerJ. 2023;11:e15678. 10.7717/peerj.15678 PMC10349557 37456882

[4] Tsakiridis T, Pond GR, Wright J, Ellis PM, Ahmed N, Abdulkarim B, et al. Metformin in combination with chemoradiotherapy in locally advanced non-small cell lung cancer: The OCOG-ALMERA randomized clinical trial. JAMA Oncol. 2021;7(9):1333–41. 10.1001/jamaoncol.2021.2328 PMC8323053 34323924

[5] Sun S, Han Q, Liang M, Zhang Q, Zhang J, Cao J. Downregulation of m(6)A reader YTHDC2 promotes tumor progression and predicts poor prognosis in non-small cell lung cancer. Thorac Cancer. 2020;11(11):3269–79. 10.1111/1759-7714.13667 PMC7606000 32956555

[6] Fang M, Hang Q, Jiang H, Cai L, Hu J, Ying H, et al. Efficacy and safety evaluation of neoadjuvant immunotherapy plus chemotherapy for resectable non-small cell lung cancer in real world. Front Oncol. 2022;12:1055610. 10.3389/fonc.2022.1055610 PMC9877512 36713546

[7] Fan X, Yang H, Zhao C, Hu L, Wang D, Wang R, et al. Local anesthetics impair the growth and self-renewal of glioblastoma stem cells by inhibiting ZDHHC15-mediated GP130 palmitoylation. Stem Cell Res Ther. 2021;12(1):107. 10.1186/s13287-021-02175-2 PMC7863430 33541421

[8] Liu H, Dilger JP, Lin J. Effects of local anesthetics on cancer cells. Pharmacol Ther. 2020;212:107558. 10.1016/j.pharmthera.2020.107558 32343985

[9] Sun D, Li YC, Zhang XY. Lidocaine promoted ferroptosis by targeting miR-382-5p/SLC7A11 axis in ovarian and breast cancer. Front Pharmacol. 2021;12:681223. 10.3389/fphar.2021.681223 PMC8188239 34122108

[10] Inchiosa MA Jr. Anti-tumor activity of phenoxybenzamine and its inhibition of histone deacetylases. PLoS One. 2018;13(6):e0198514. 10.1371/journal.pone.0198514 PMC5999115 29897996

[11] Gu JH, Liu CC, Xie JL, Ma B, Cui SM, Yang GZ, et al. The local anesthetic bupivacaine inhibits the progression of non-small cell lung cancer by inducing autophagy through Akt/mTOR signaling. Front Oncol. 2021;11:616445. 10.3389/fonc.2021.616445 PMC7991299 33777755

[12] Zheng X, Dong L, Zhao S, Li Q, Liu D, Zhu X, et al. Propofol affects non-small-cell lung cancer cell biology by regulating the miR-21/PTEN/AKT pathway in vitro and in vivo. Anesth Analg. 2020;131(4):1270–80. 10.1213/ANE.0000000000004778 32925348

[13] Zhang Q, Cheng F, Zhang Z, Wang B, Zhang X. Propofol suppresses non-small cell lung cancer tumorigenesis by regulation of circ-RHOT1/miR-326/FOXM1 axis. Life Sci. 2021;119042. 10.1016/j.lfs.2021.119042 33515563

[14] Huang Y, Lei L, Liu Y. Propofol improves sensitivity of lung cancer cells to cisplatin and its mechanism. Med Sci Monit. 2020;26:e919786. 10.12659/MSM.919786 PMC7142322 32225124

[15] Zhang Y, Jiang L, Ouyang J, Du X, Jiang L. Efficacy and safety of traditional Chinese medicine injections combined with FOLFOX4 regimen for gastric cancer: A protocol for systematic review and network meta-analysis. Medicine (Baltimore). 2021;100(41):e27525. 10.1097/MD.0000000000027525 PMC8519213 34731143

[16] Xiong K, Zhang Y, Wen Q, Luo J, Lu Y, Wu Z, et al. Co-delivery of paclitaxel and curcumin by biodegradable polymeric nanoparticles for breast cancer chemotherapy. Int J Pharm. 2020;589:119875. 10.1016/j.ijpharm.2020.119875 32919003

[17] Zhang X, Liang T, Yang W, Zhang L, Wu S, Yan C, et al. Astragalus membranaceus injection suppresses production of interleukin-6 by activating autophagy through the AMPK-mTOR pathway in Lipopolysaccharide-stimulated macrophages. Oxid Med Cell Longev. 2020;2020:1364147. 10.1155/2020/1364147 PMC7364262 32724488

[18] Sheng Z, Jiang Y, Liu J, Yang B. UHPLC-MS/MS analysis on flavonoids composition in astragalus membranaceus and their antioxidant activity. Antioxidants (Basel). 2021;10(11):1852. 10.3390/antiox10111852 PMC8614773 34829723

[19] Wu TH, Yeh KY, Wang CH, Wang H, Li TL, Chan YL, et al. The combination of astragalus membranaceus and angelica sinensis inhibits lung cancer and cachexia through its immunomodulatory function. J Oncol. 2019;2019:9206951. 10.1155/2019/9206951 PMC6875282 31781219

[20] Zang Y, Wan J, Zhang Z, Huang S, Liu X, Zhang W. An updated role of astragaloside IV in heart failure. Biomed Pharmacother. 2020;126:110012. 10.1016/j.biopha.2020.110012 32213428

[21] Jia L, Lv D, Zhang S, Wang Z, Zhou B. Astragaloside IV inhibits the progression of non-small cell lung cancer through the Akt/GSK-3β/β-catenin pathway. Oncol Res. 2019;27(4):503–8. 10.3727/096504018X15344989701565 PMC7848426 30131090

[22] Lai ST, Wang Y, Peng F. Astragaloside IV sensitizes non-small cell lung cancer cells to cisplatin by suppressing endoplasmic reticulum stress and autophagy. J Thorac Dis. 2020;12(7):3715–24. 10.21037/jtd-20-2098 PMC7399439 32802451

[23] Ling Q, Wu S, Liao X, Liu C, Chen Y. Anesthetic propofol enhances cisplatin-sensitivity of non-small cell lung cancer cells through N6-methyladenosine-dependently regulating the miR-486-5p/RAP1-NF-kappaB axis. BMC Cancer. 2022;22(1):765. 10.1186/s12885-022-09848-y PMC9281112 35836137

[24] Liu Y, Wu L, Ao H, Zhao M, Leng X, Liu M, et al. Prognostic implications of autophagy-associated gene signatures in non-small cell lung cancer. Aging (Albany NY). 2019;11(23):11440–62. 10.18632/aging.102544 PMC6932887 31811814

[25] Zhang XW, Liu W, Jiang HL, Mao B. Chinese herbal medicine for advanced non-small-cell lung cancer: A systematic review and meta-analysis. Am J Chin Med. 2018;46(5):923–52. 10.1142/S0192415X18500490 30001642

[26] Yang J, Zhu X, Yuan P, Liu J, Wang B, Wang G. Efficacy of traditional Chinese Medicine combined with chemotherapy in patients with non-small cell lung cancer (NSCLC): a meta-analysis of randomized clinical trials. Support Care Cancer. 2020;28(8):3571–9. 10.1007/s00520-020-05433-w 32266566

[27] Zeng J, Li YK, Quan FF, Zeng X, Chen CY, Zeng T, et al. Propofol‑induced miR‑125a‑5p inhibits the proliferation and metastasis of ovarian cancer by suppressing LIN28B. Mol Med Rep. 2020;22(2):1507–17. 10.3892/mmr.2020.11223 PMC7346589 32627014

[28] Yu X, Gao Y, Zhang F. Propofol inhibits pancreatic cancer proliferation and metastasis by up-regulating miR-328 and down-regulating ADAM8. Basic Clin Pharmacol Toxicol. 2019;125(3):271–8. 10.1111/bcpt.13224 30861616

[29] Du Y, Zhang X, Zhang H, Chen Y, Zhu S, Shu J, et al. Propofol modulates the proliferation, invasion and migration of bladder cancer cells through the miR‑145‑5p/TOP2A axis. Mol Med Rep. 2021;23(6):439. 10.3892/mmr.2021.12078 PMC8060790 33846791

[30] Liang B, Dong T. Effects of propofol on invasion and migration of colon cancer cells and JAK2/STAT3 signaling pathway. Zhong Nan Da Xue Xue Bao Yi Xue Ban. 2020;45(3):290–6. 10.11817/j.issn.1672-7347.2020.180704 32386021

[31] Xing SG, Zhang KJ, Qu JH, Ren YD, Luan Q. Propofol induces apoptosis of non-small cell lung cancer cells via ERK1/2-dependent upregulation of PUMA. Eur Rev Med Pharmacol Sci. 2018;22(13):4341–9. 10.26355/eurrev_201807_15431 30024623

[32] Bayat Mokhtari R, Homayouni TS, Baluch N, Morgatskaya E, Kumar S, Das B, et al. Combination therapy in combating cancer. Oncotarget. 2017;8(23):38022–43. 10.18632/oncotarget.16723 PMC5514969 28410237

[33] Shirbhate E, Patel P, Patel VK, Veerasamy R, Sharma PC, Rajak H. The combination of histone deacetylase inhibitors and radiotherapy: a promising novel approach for cancer treatment. Future Oncol. 2020;16(30):2457–69. 10.2217/fon-2020-0385 32815411

[34] Satoh H, Kagohashi K. Response to erlotinib and bevacizumab combination therapy after acquired resistance to osimertinib in patients with non-small cell lung cancer. Anticancer Drugs. 2022;33(3):320–2. 10.1097/CAD.0000000000001142 34974477

[35] Gao J, Ding C, Zhou J, Wu G, Han Z, Li J, et al. Propofol suppresses lung cancer tumorigenesis by modulating the circ-ERBB2/miR-7-5p/FOXM1 axis. Thorac Cancer. 2021;12(6):824–34. 10.1111/1759-7714.13856 PMC7952809 33506582

[36] Yang N, Liang Y, Yang P, Ji F. Propofol suppresses LPS-induced nuclear accumulation of HIF-1alpha and tumor aggressiveness in non-small cell lung cancer. Oncol Rep. 2017;37(5):2611–9. 10.3892/or.2017.5514 PMC5428906 28426124

[37] Xu F, Cui WQ, Wei Y, Cui J, Qiu J, Hu LL, et al. Astragaloside IV inhibits lung cancer progression and metastasis by modulating macrophage polarization through AMPK signaling. J Exp Clin Cancer Res. 2018;37(1):207. 10.1186/s13046-018-0878-0 PMC6116548 30157903

[38] Quan Y, Li S, Wang Y, Liu G, Lv Z, Wang Z. Propofol and sevoflurane alleviate malignant biological behavior and cisplatin resistance of Xuanwei lung adenocarcinoma by modulating the Wnt/β-catenin pathway and PI3K/AKT pathway. Anticancer Agents Med Chem. 2022;22(11):2098–108. 10.2174/1871520621666211026092405 35152870

[39] Yang X, Qin J, Gong C, Yang J. Propofol enhanced the cell sensitivity to paclitaxel (PTX) in prostatic cancer (PC) through modulation of HOTAIR. Genes Genomics. 2021;43(7):807–14. 10.1007/s13258-021-01093-0 33893626

[40] Zhang S, Tang D, Zang W, Yin G, Dai J, Sun YU, et al. Synergistic inhibitory effect of traditional chinese medicine astragaloside IV and curcumin on tumor growth and angiogenesis in an orthotopic nude-mouse model of human hepatocellular carcinoma. Anticancer Res. 2017;37(2):465–73. 10.21873/anticanres.11338 28179291

[41] Li YJ, Lei YH, Yao N, Wang CR, Hu N, Ye WC, et al. Autophagy and multidrug resistance in cancer. Chin J Cancer. 2017;36(1):52. 10.1186/s40880-017-0219-2 PMC5482965 28646911

[42] Amaravadi R, Kimmelman AC, White E. Recent insights into the function of autophagy in cancer. Genes Dev. 2016;30(17):1913–30. 10.1101/gad.287524.116 PMC5066235 27664235

[43] Zhang YF, Li CS, Zhou Y, Lu XH. Propofol facilitates cisplatin sensitivity via lncRNA MALAT1/miR-30e/ATG5 axis through suppressing autophagy in gastric cancer. Life Sci. 2020;244:117280. 10.1016/j.lfs.2020.117280 31926239

[44] Zhao Y, Wang L, Wang Y, Dong S, Yang S, Guan Y, et al. Astragaloside IV inhibits cell proliferation in vulvar squamous cell carcinoma through the TGF-β/SMAD signaling pathway. Dermatol Ther. 2019;32(4):e12802. 10.1111/dth.12802 30536730

